# Paradoxical control of multifocal mammary oncogenesis by radiation therapy

**DOI:** 10.1080/2162402X.2025.2458886

**Published:** 2025-01-28

**Authors:** Lorenzo Galluzzi, Aitziber Buqué

**Affiliations:** Cancer Signaling and Microenvironment Program, Fox Chase Cancer Center, Philadelphia, PA, USA

**Keywords:** CD4^+^CD25^+^FOXP3^+^ T_REG_ cells, CTLA4, dendritic cells, IL-1β, immunotherapy, PD-1

## Abstract

In an immunocompetent mouse model of multifocal, metachronous HR^+^ mammary carcinogenesis, we have recently demonstrated that a superior control of primary neoplastic lesions by focal radiotherapy does not necessarily translate into improved oncosuppression at non-irradiated (pre)malignant tissues. These data point to a link between local tumor control by radiotherapy and systemic oncogenesis that remains to be fully understood.

Hormone receptor (HR)^+^ breast cancer is widely considered as an immunologically ‘cold’ tumor as it exhibits limited infiltration by immune effectors including dendritic cells (DCs), CD8^+^ cytotoxic T lymphocytes (CTLs) and natural killer (NK) cells at baseline.^1,2^ In line with this notion, patients with HR^+^ breast cancer are poorly responsive to immune checkpoint inhibitors (ICIs) targeting programmed cell death 1 (PDCD1, best known as PD-1) employed as standalone immunotherapeutic interventions.^3^ Thus, clinically viable approaches that may inflame the tumor microenvironment (TME) in patients with HR^+^ breast cancer to convert their malignancy into an immunologically ‘hot’ disease that responds to ICIs are warranted.^1,4^

For over a decade now, focal radiation therapy (RT) has attracted considerable attention as a means to convert immunologically cold and hence ICI-resistant tumors into hot lesions that respond to ICIs, overall reflecting a well-defined and predictable safety profile, widespread geographical availability, and a non-negligible potential for immunostimulation (at least in some settings, contingent on dose, fractionation schedule and target volume).^5–8^ In this context, we aimed at evaluating whether focal RT can be successfully combined with ICIs and other forms of immunotherapy to generate robust anticancer immune responses with systemic oncosuppressive activity. To this objective, we harnessed a mouse model of multifocal, metachronous HR^+^ mammary carcinogenesis with unique resemblance to human HR^+^ breast cancer – namely HR^+^ mammary tumorigenesis as elicited in immunocompetent female C57BL/6 mice by slow-release medroxyprogesterone acetate (MPA, M) pellets and oral dimethylbenz[α]anthracene (DMBA, D)^9^ – to investigate the effects of focal RT delivered to the first detectable M/D-driven tumor (optionally in combination with otherwise systemically inactive immunotherapeutics) on the emergence and growth of new neoplastic lesions at non-irradiated mammary tissues. Focal RT to the first detectable tumor invariably prolonged the overall survival (OS) of mice bearing M/D-driven mammary carcinomas (to an extent that depended on RT dose and fractionation schedule). However, beyond a hitherto poorly defined threshold, achieving superior tumor control at irradiated disease sites did not necessarily result into an extra OS benefit, owing to the accelerated development of new neoplastic lesions at non-irradiated (pre)malignant tissues.^10^ Our findings point to a connection between local tumor control by focal RT and systemic oncogenesis that has not yet been experimentally dissected.

Initially, we set out to check the ability of focal hypofractionated RT delivered in various doses and fractionation schedules to control the growth of established M/D-driven mammary carcinomas, finding (amongst the RT regimens tested) an optimal effect for 2 fractions of 20 Gy each delivered on consecutive days (which resulted in the complete eradication of irradiated lesions in 90% of mice). The OS benefit afforded by the 20 Gy X 2 regimen, however, was virtually equivalent to that afforded by an 8 Gy X 6 regimen (despite this being ablative only in < 20% of mice), at least in part reflecting: (1) decelerated oncogenesis and/or tumor progression at non-irradiated (pre)malignant sites in the latter *vs* the former setting, and (2) the contribution of all palpable neoplastic lesions to cumulative tumor burden (which defines humane endpoint).^10^

Interestingly, combining these two RT regimens with a monoclonal antibody specific for PD-1 (which *per se* mediates marginal anticancer effects in this tumor model) failed to significantly alter OS extension as provided by RT alone, but considerably changed the relative contribution of primary (irradiated) vs secondary (non-irradiated) disease to cumulative tumor burden at humane endpoint. Thus, while adding a PD-1 blocker to focal RT delivered in 6 fractions of 8 Gy each improved primary disease control, it also accelerated oncogenesis/disease progression at non-irradiated (pre)malignant sites, overall resulting in comparable OS. Similar findings were obtained by combining a PD-1 blocker plus recombinant fms related receptor tyrosine kinase 3 ligand (FLT3LG) – together mediating a minimal activity on tumor growth and OS – with focal RT delivered in 3 fractions of 10 Gy. Instead, the contrary was true when the 10 Gy X 3 RT regimen was combined with an otherwise inactive interleukin 1 beta (IL1B, best known as IL-1β) blocker. Indeed, the local efficacy of focal RT delivered in 3 fractions of 10 Gy each was reduced by the systemic administration of an IL-1β blocker, but this was accompanied by decelerated oncogenesis/disease progression at non-irradiated (pre)malignant sites, with these two (immuno)therapeutic combinatorial regimens providing a similar OS benefit to mice bearing M/D-driven mammary carcinomas. Similar results were obtained by combining the 20 Gy X 2 RT regimen with a monoclonal antibody specific for PD-1.^10^

In summary, our findings suggest the existence of a hitherto poorly delineated connection between local cancer control by focal RT and systemic oncogenesis and tumor progression at non-irradiated (pre)malignant disease sites. More specifically, at least in our immunocompetent model of multifocal, metachronous HR^+^ carcinogenesis, pushing local disease control by RT beyond a largely undefined threshold (by changing dose and fractionation regimen, or by combining RT with immunotherapy), appears to provide no added OS survival benefits, mostly reflecting the accelerated emergence and/or growth of new, non-irradiated neoplastic lesions (Figure 1). Given its sensitivity to (otherwise systemically inactive) immunomodulators, we surmise that such a link may be immunological in nature, despite the fact that different malignant lesions as emerging in female C57BL/6 mice subjected to M/D-driven carcinogenesis *de facto* constitute independent tumors, and hence are not expected to share considerable antigenic similarities beyond common mammary antigens. The potential implication of tissue-specific (rather than tumor-specific) antigens in our observations is supported by the fact that M/D-driven carcinogenesis in mice can be effectively delayed by administering non-transformed mammary organoids subjected to RT *in vitro* during the oncogenic process.^9^ Additional work, however, is required to formally validate this possibility.

**Figure 1.**
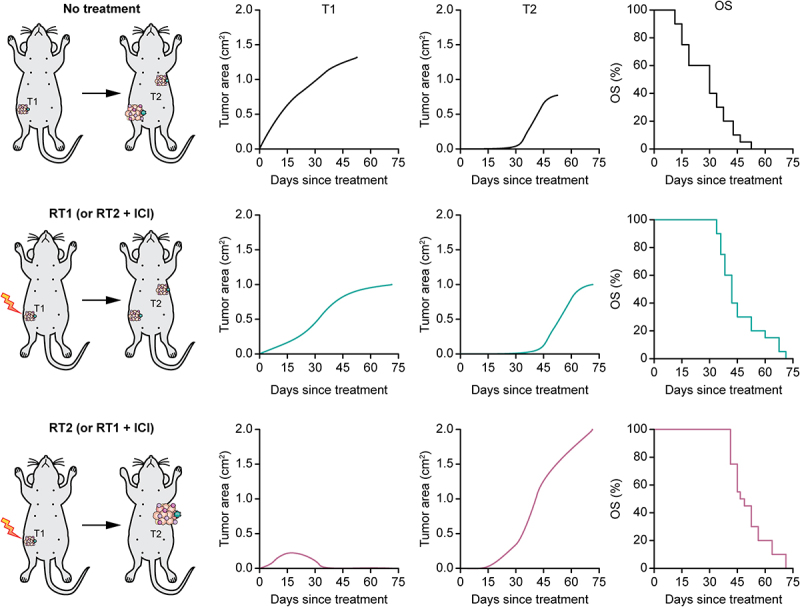
Local disease control RT vs distant oncosuppression. In an immunocompetent mouse model of multifocal, metachronous HR^+^ mammary carcinogenesis, we found that ameliorating local disease control by focal hypofractionated radiation therapy (RT) beyond a poorly understood threshold appears to confer no extra overall survival (OS) benefit to tumor-bearing mice, mostly reflecting limited oncosuppression at distant, non-irradiated disease sites. At least in this model, indeed, untreated mice mostly need to be euthanatized because of the uncontrolled growth of the first detectable tumor (T1), with a limited contribution to global disease burden at humane endpoint from lesions appearing later (T2). However, the OS extension provided by delivering hypofractionated RT to T1 (or RT to T1 plus an otherwise inactive immune checkpoint inhibitor, ICI) – when these regimens enable sizable but incomplete local disease control in the absence of measurable alterations of T2 appearance and growth (RT1 or RT2 + ICI) – cannot be improved by increasing RT dose or combining it with a distinct (but also otherwise inactive) ICI resulting in superior T1 control (RT2 or RT1 + ICI), owing to accelerated T2 development. Please note that tumor growth and OS graphs depicted here are for exemplificatory purposes only and do not illustrate actual experimental data.

In the future, it will be important to understand whether similar observations can be extrapolated to *bona fide* metastatic tumor models. In such case, indeed, achieving complete local control by RT may not always represent an optimal strategy to unlock clinical responses to ICIs at non-irradiated disease sites in patients with ICI-resistant tumors.
